# *Micromachines* 2022 Best Paper Awards

**DOI:** 10.3390/mi13060858

**Published:** 2022-05-30

**Authors:** Micromachines Editorial Office

**Affiliations:** MDPI, St. Alban-Anlage 66, 4052 Basel, Switzerland; micromachines@mdpi.com

*Micromachines* is instituting the Best Paper Awards to recognize outstanding papers published in the journal. We are now pleased to announce the winners of the “Micromachines 2022 Best Paper Awards”.

Papers published in 2020 were preselected by the *Micromachines* Editorial Office on the basis of the number of citations and downloads from the website. The winners from the nominations were determined by an award committee, the Editor-in-Chief, together with the Editorial Office. The following five top-voted papers, in no particular order, have won the *Micromachines* 2022 Best Paper Awards:

**1.**  **Research Article**

**Size Sorting of Exosomes by Tuning the Thicknesses of the Electric Double Layers on a Micro-Nanofluidic Device**

By Satoko Fujiwara, Kyojiro Morikawa, Tatsuro Endo, Hideaki Hisamoto and Kenji Sueyoshi (Figure 1).

*Micromachines* 2020, 11(5), 458; https://doi.org/10.3390/mi11050458.

Available online: https://www.mdpi.com/2072-666X/11/5/458.

Synopsis of the paper by the authors:

Exosomes, a type of extracellular vesicle with a diameter of 30–150 nm, perform key biological functions such as intercellular communication. Recently, size sorting of exosomes has received increasing attention in order to clarify the correlation between their size and components. However, such sorting remains extremely difficult. Here, we propose to sort their size by controlling their electrokinetic migration in nanochannels in a micro-nanofluidic device, which is achieved by tuning the thickness of the electric double layers in the nanochannels. This approach was demonstrated experimentally for exosomes smaller than 250 nm. Using different running-buffer concentrations (1 × 10^−3^, 1 × 10^−4^, and 1 × 10^−5^ M), most of the exosomes larger than 140, 110, and 80 nm were successfully cut off downstream of the nanochannels, respectively. Therefore, the proposed method is applicable for the size sorting of exosomes [1].

**2.**  **Research Article**

**Additive Manufacturing of Sub-Micron to Sub-mm Metal Structures with Hollow AFM Cantilevers**

By Giorgio Ercolano, Cathelijn van Nisselroy, Thibaut Merle, János Vörös, Dmitry Momotenko, Wabe W. Koelmans and Tomaso Zambelli (Figure 2).

*Micromachines* 2020, 11(1), 6; https://doi.org/10.3390/mi11010006.

Available online: https://www.mdpi.com/2072-666X/11/1/6.

Synopsis of the paper by the authors:

We describe our force-controlled 3D-printing method for layer-by-layer additive micromanufacturing (µAM) of metal microstructures. Hollow atomic force microscopy cantilevers are utilized to locally dispense metal ions in a standard three-electrode electrochemical cell, enabling a confined electroplating reaction. The deflection feedback signal enables the live monitoring of the voxel growth and the consequent automation of the printing protocol in a layer-by-layer fashion for the fabrication of arbitrary-shaped geometries. In a second step, we investigated the effect of the free parameters (aperture diameter, applied pressure, and applied plating potential) on the voxel size, which enabled us to tune the voxel dimensions on the fly, as well as to produce objects spanning at least two orders of magnitude in each direction. As a concrete example, we printed two different replicas of Michelangelo’s David. Copper was used as the metal, but the process can in principle be extended to all metals that are macroscopically electroplated in a standard way [2].

**3.**  **Research Article**

**A Robotic Biopsy Endoscope with Magnetic 5-DOF Locomotion and a Retractable Biopsy Punch**

By Manh Cuong Hoang, Viet Ha Le, Kim Tien Nguyen, Van Du Nguyen, Jayoung Kim, Eunpyo Choi, Seungmin Bang, Byungjeon Kang, Jong-Oh Park and Chang-Sei Kim (Figure 3).

*Micromachines* 2020, 11(1), 98; https://doi.org/10.3390/mi11010098.

Available online: https://www.mdpi.com/2072-666X/11/1/98.

Synopsis of the paper by the authors:

Capsule endoscopes (CEs) have emerged as an advanced diagnostic technology for gastrointestinal diseases in recent decades. However, with regard to robotic motions, they require active movability and multi-functionalities for extensive, untethered, and precise clinical utilization. Herein, we present a novel wireless biopsy CE employing active five-degree-of-freedom locomotion and a biopsy-needle punching mechanism for the histological analysis of the intestinal tract. A medical biopsy punch is attached to a screw mechanism, which can be magnetically actuated to extrude and retract the biopsy tool for tissue extraction. The external magnetic field from an electromagnetic actuation (EMA) system is utilized to actuate the screw mechanism and harvest biopsy tissue; therefore, the proposed system consumes no onboard energy of the CE. This design enables the observation of the biopsy process through the capsule’s camera. A prototype with a diameter of 12 mm and length of 30 mm was fabricated with a medical biopsy punch with a diameter of 1.5 mm. Its performance was verified through numerical analysis, as well as in vitro and ex vivo experiments on porcine intestine. The CE could be moved to target lesions and obtain sufficient tissue samples for histological examination. The proposed biopsy CE mechanism utilizing punch biopsy and its wireless extraction–retraction technique could advance untethered intestinal endoscopic capsule technology at clinical sites [3].

**4.**  **Research Article**

**Development of Fully Flexible Tactile Pressure Sensor with Bilayer Interlaced Bumps for Robotic Grasping Applications**

By Lingfeng Zhu, Yancheng Wang, Deqing Mei and Chengpeng Jiang (Figure 4).

*Micromachines* 2020, 11(8), 770; https://doi.org/10.3390/mi11080770.

Available online: https://www.mdpi.com/2072-666X/11/8/770.

Synopsis of the paper by the authors:

Flexible tactile sensors have been utilized in intelligent robotics for human–machine interaction and healthcare monitoring. The relatively low flexibility, unbalanced sensitivity, and sensing range of the tactile sensors hinder the accurate tactile information perception during robotic hand grasping of different objects. This paper developed a fully flexible tactile pressure sensor using flexible graphene and silver composites as the sensing element and stretchable electrodes, respectively. As for the structural design of the tactile sensor, the proposed bilayer interlaced bumps can be used to convert external pressure into the stretching of graphene composites. The fabricated tactile sensor exhibits high sensing performance, including relatively high sensitivity (up to 3.40% kPa^−1^), a wide sensing range (200 kPa), a good dynamic response, and considerable repeatability. Then, the tactile sensor was integrated with the robotic hand finger, and the grasping results indicated the capability of using the tactile sensor to detect the distributed pressure during grasping applications. The grasping motions and properties of the objects can be further analyzed through the acquired tactile information in time and spatial domains, demonstrating the potential applications of the tactile sensor in intelligent robotics and human–machine interfaces [4].

**5.**  **Research Article**

**A mm-Sized Free-Floating Wireless Implantable Opto-Electro Stimulation Device**

By Yaoyao Jia, Yan Gong, Arthur Weber, Wen Li and Maysam Ghovanloo (Figure 5).

*Micromachines* 2020, 11(6), 621; https://doi.org/10.3390/mi11060621.

Available online: https://www.mdpi.com/2072-666X/11/6/621.

Synopsis of the paper by the authors:

Towards a distributed neural interface, consisting of multiple miniaturized implants, for interfacing with large-scale neuronal ensembles over large brain areas, this paper presents a mm-sized free-floating wirelessly powered implantable opto-electro stimulation (FF-WIOS2) device equipped with a 16-channel optical and 4-channel electrical stimulation for reconfigurable neuromodulation. The FF-WIOS2 is wirelessly powered and controlled through a three-coil inductive link at 60 MHz. The FF-WIOS2 receives stimulation parameters via an on–off keying (OOK) while sending its rectified voltage information to an external headstage for closed-loop power control (CLPC) via a load-shift keying (LSK). The FF-WIOS2 system-on-chip (SoC), fabricated using a 0.35 µm standard CMOS process, employs switched-capacitor-based stimulation (SCS) architecture to provide the large instantaneous current needed for surpassing the optical stimulation threshold. The SCS charger charges an off-chip capacitor up to 5 V at 37% efficiency. At the onset of stimulation, the capacitor delivers charge with a peak current in the 1.7–12 mA range to a micro-LED (µLED) array for optical stimulation or 100–700 μA range to a micro-electrode array (MEA) for biphasic electrical stimulation. Active and passive charge-balancing circuits are activated in electrical stimulation mode to ensure stimulation safety. In vivo experiments conducted on three anesthetized rats verified the efficacy of the two stimulation mechanisms. The proposed FF-WIOS2 is potentially a reconfigurable tool for performing untethered neuromodulation [5].

These five papers represent valuable contributions to *Micromachines*. We warmly congratulate both teams on their accomplishments and wish them continued success.

*Micromachines* 2022 Best Paper Awards Committee,

*Micromachines* Editorial Board.

## Figures and Tables

**Figure 1:**
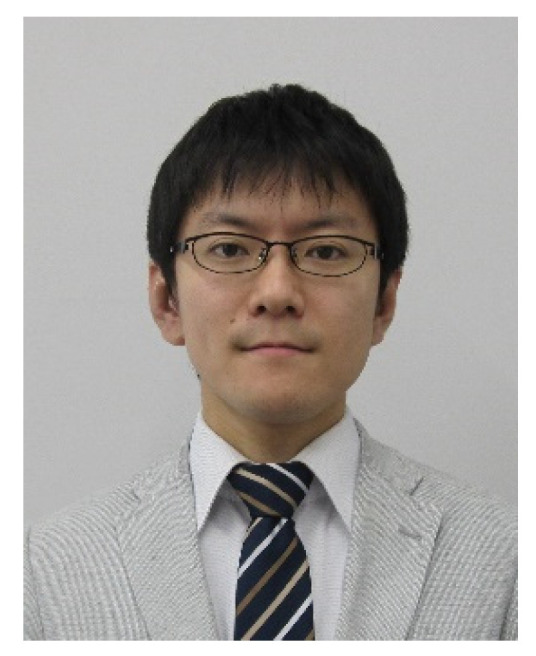
Kenji Sueyoshi.

**Figure 2:**
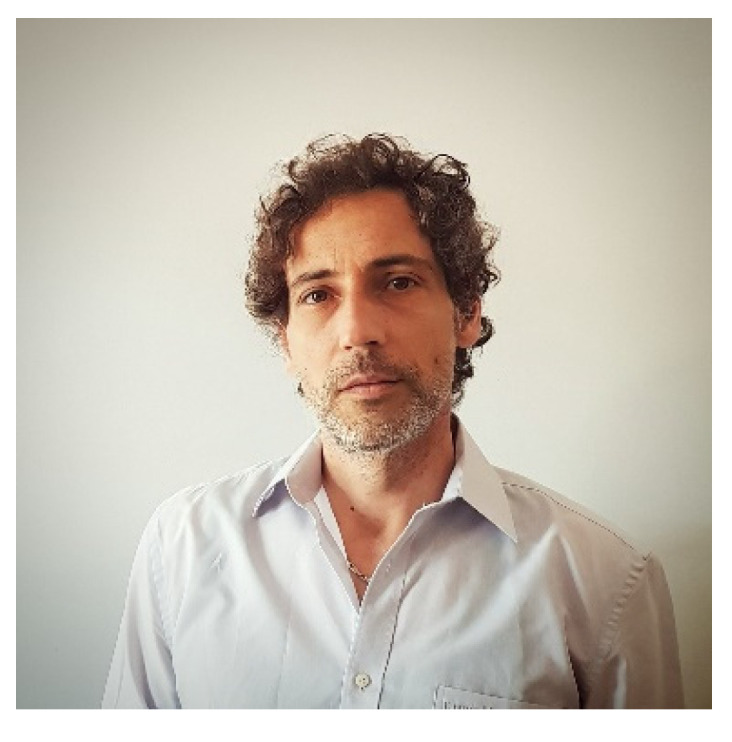
Giorgio Ercolano.

**Figure 3:**
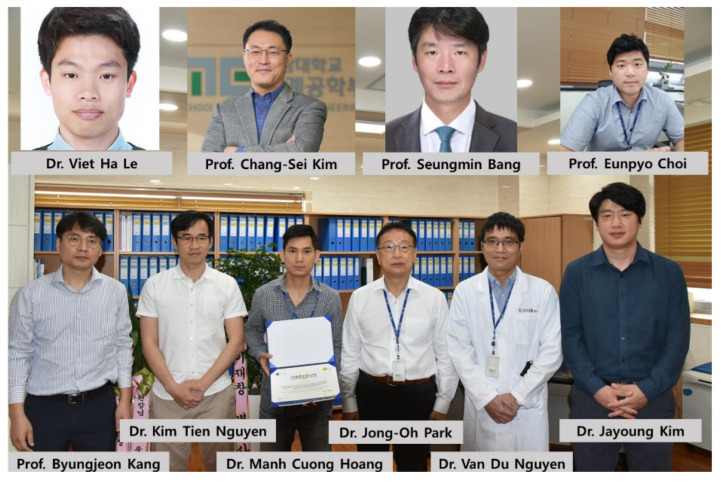
Chang-Sei Kim Team.

**Figure 4:**
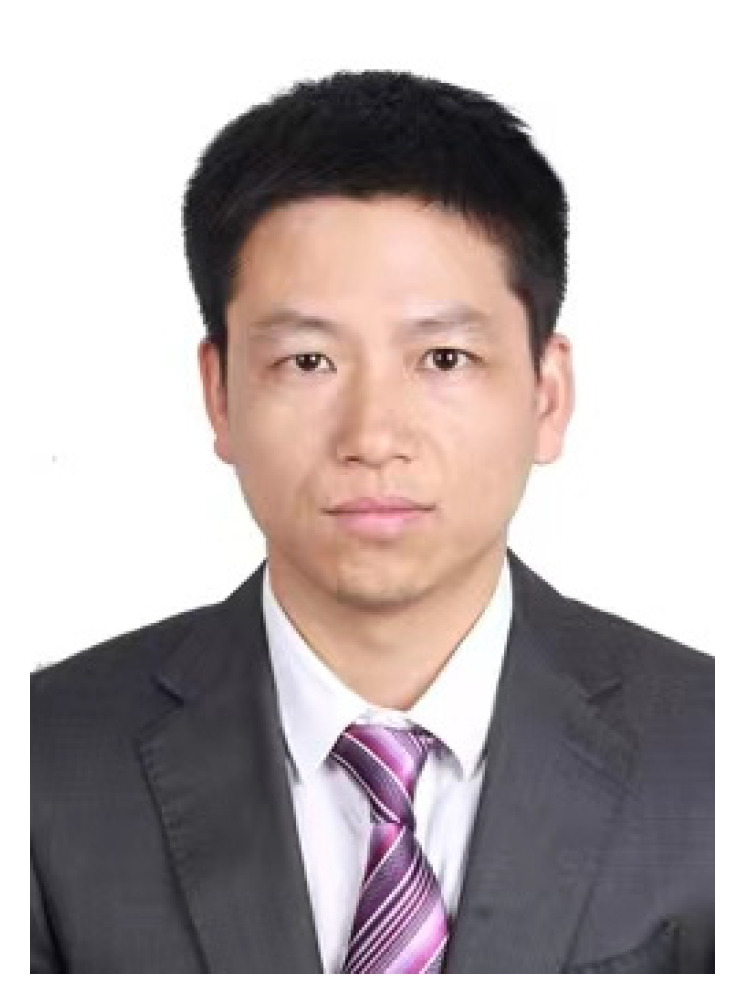
Yancheng Wang.

**Figure 5:**
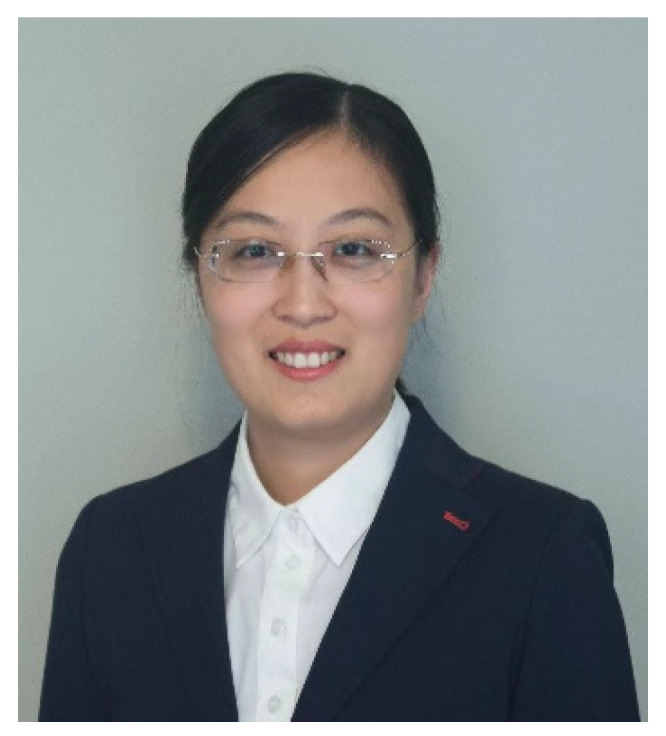
Yaoyao Jia.
